# Identification of prognostic immune-related genes in the tumor microenvironment of endometrial cancer

**DOI:** 10.18632/aging.102817

**Published:** 2020-02-19

**Authors:** Peigen Chen, Yuebo Yang, Yu Zhang, Senwei Jiang, Xiaomao Li, Jing Wan

**Affiliations:** 1 Department of Gynecology, The Third Affiliated Hospital of Sun Yat-Sen University, Guangzhou, Guangdong Province, China

**Keywords:** endometrial cancer, tumor microenvironment, prognosis, immune score, TCGA

## Abstract

Endometrial cancer (EC) is one of the most common gynecologic malignancies. To identify potential prognostic biomarkers for EC, we analyzed the relationship between the EC tumor microenvironment and gene expression profiles. Using the ESTIMATE R tool, we found that immune and stromal scores correlated with clinical data and the prognosis of EC patients. Based on the immune and stromal scores, 387 intersection differentially expressed genes were identified. Eight immune-related genes were then identified using two machine learning algorithms. Functional enrichment analysis revealed that these genes were mainly associated with T cell activation and response. Kaplan-Meier survival analysis showed that expression of TMEM150B, CACNA2D2, TRPM5, NOL4, CTSW, and SIGLEC1 significantly correlated with overall survival times of EC patients. In addition, using the TIMER algorithm, we found that expression of TMEM150B, SIGLEC1, and CTSW correlated positively with the tumor infiltration levels of B cells, CD8+ T cells, CD4+ T cells, macrophages, and dendritic cells. These findings indicate that the composition of the tumor microenvironment affects the clinical outcomes of EC patients, and suggests that it may provide a basis for development of novel prognostic biomarkers and immunotherapies for EC patients.

## INTRODUCTION

Endometrial cancer (EC) is one of the most common gynecologic malignancies, and the fourth most common cancer (about 4.8% of all cancers) in women [1]. EC affects mainly post-menopausal women [2]. The routine treatment for EC includes surgery, radiotherapy, chemotherapy, and hormonal therapy. When the disease is confined to the uterus, EC patients have a relatively good prognosis, with a 5-year survival rate of 95 %. However, when distant metastases are present at the time of diagnosis, the 5-year survival rate is only 17 %, and patients respond poorly to conventional therapies. Thus, it is critical to identify prognostic biomarkers for EC, and develop therapies that are more effective for patients with advanced forms of EC.

Tumor microenvironment (TME), the site where the tumor is located, consists of immune cells, mesenchymal cells, endothelial cells, inflammatory mediators, and extracellular matrix (ECM) molecules [3, 4]. TME has a significant impact on tumor growth, chemoresistance, and clinical outcomes [5–9]; however, relatively little is known about the impact of TME on endometrial cancer. Infiltrating stromal and immune cells are the major components of TME, and play an essential role in cancer biology. The ESTIMATE (Estimation of STromal and Immune cells in MAlignant Tumor tissues using Expression data) algorithm uses gene expression data to estimate the levels of infiltrating stromal and immune cells, and tumor purity. The predictive ability of this method has been validated in large and independent datasets [6].

Machine learning is a form of artificial intelligence that can automatically analyze patterns from sample data, and make corresponding predictions. Due to its accuracy and predictive performance, the machine learning algorithm is used in different fields, including medical diagnostics [10]. The commonly used machine learning algorithms include SVM, KNN, LASSO, and Random forest.

Knowledge of the TME composition is critical to understand the interactions between cancer and immune cells, and the impact the immune system has on tumor behavior [11]. In this study, we used the ESTIMATE and TIMER (Tumor Immune Estimation Resource) [12] algorithms, to perform a comprehensive analysis of immune cells and genes in the TME of endometrial carcinoma, and to correlate the data to clinical outcomes and prognosis of EC patients. Our results indicate that the TME composition affects the clinical outcomes of EC patients, suggesting that it might provide a basis for development of new prognostic biomarkers and therapies, especially immunotherapies, for EC patients.

## RESULTS

### Immune and stromal scores correlate with EC clinical data and prognosis

In total, data from 521 EC patients, and 19459 RNAs extracted from RNA-seq data according to ENSEMBL Genomes (hg38), were analyzed in this study. Based on the gene expression data, immune and stromal scores were calculated using the ESTIMATE algorithm (Supplementary Table 1).

**Figure 1:**
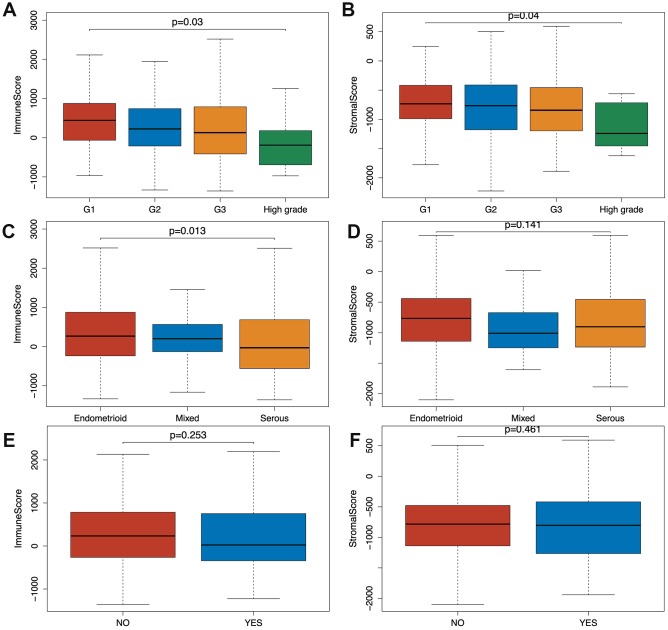
**Relationship between immune and stromal scores and EC clinical and pathological data.** (**A**, **B**) Distribution of immune and stromal scores of EC grades. (**C, D**) Distribution of immune and stromal scores of EC pathologic type, including endometrioid cancer, serous cancer and mix type. (**E**, **F**) Distribution of immune and stromal scores of new tumor event after initial treatment of EC.

Based on the clinical data extracted from TCGA-CDR (Supplementary Table 2) and using Wilcoxon signed-rank test, we found that both immune and stromal scores of grade 3 (G3; n=319) and high-grade (n=11) EC were significantly lower compared to grade 1 (G1; n=96) and grade 2 (G2; n=119) groups (*p*=0.03, *p*=0.04). In addition, the scores of high grade patients were lower than the scores of grade 3 patients (*p*=0.03, *p*=0.04) (Figure 1A, 1B). Based on a classification by the International Society of Gynecological Pathologists, the clinical outcomes of grade 1 and grade 2 patients are better than grade 3 and high grade patients, and the high grade patients have even worse prognosis than the grade 3 patients [13]. As shown in Figure 1C and 1D, the immune and stromal scores were associated with the EC pathological subtype: endometrioid endometrial adenocarcinoma had higher immune and stromal scores than serous endometrial adenocarcinoma. In addition, when we compared the immune and stromal scores between patients with a new tumor event (n= 115) and without new tumor event (n= 370) after initial treatment, patients without a new tumor event had higher immune and stromal scores, although this did not reach a statistical significance (Figure 1E, 1F).

**Figure 2:**
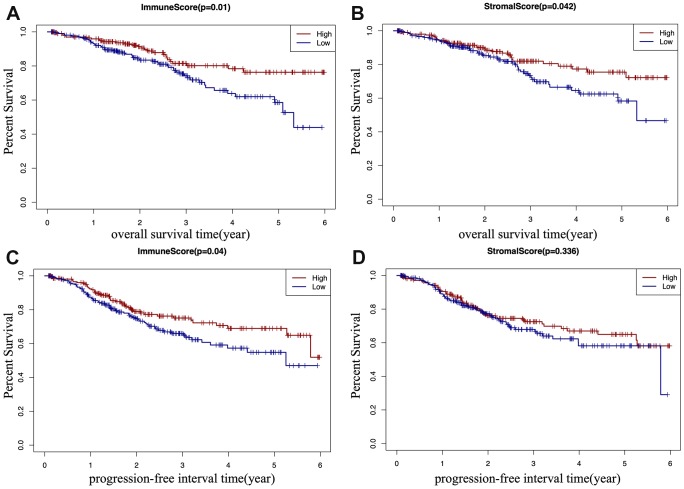
**Kaplan-Meier (KM) survival curve of EC patients based on their immune/stromal scores.** Patients were classified into high immune/stromal scores groups and low immune/stromal scores groups. (**A**) The KM curve of overall survival (OS) time of high and low immune score group. (**B**) The KM curve of OS time of high and low stromal score group. (**C**) The KM curve of progression-free interval (PFI) time according to immune scores. (**D**) The KM curve of progression-free interval (PFI) time according to stromal scores.

According to TCGA-CDR, a progression-free interval (PFI) is defined as the time until patients develop a new tumor event, including recurrence of disease and distant metastasis [14]. To determine whether there is a correlation between the immune and stromal scores, the overall survival (OS) time, and the PFI of EC patients (Figure 2A–2D), EC patients were classified into a high score group (n(stromal group) =243, n(immune group)= 242), and a low score group (n(stromal group) =242, n(immune group)= 243) based on the median of scores, and Kaplan–Meier survival curve was used to analyze the correlation. We found that the high immune score positively correlated with both OS (*p*=0.01) and PFI (*p*=0.04) (Figure 2A, 2C), and the high stromal score positively correlated with OS (*p*=0.042) (Figure 2B).

### Identification of differentially expressed genes (DEGs)

**Figure 3:**
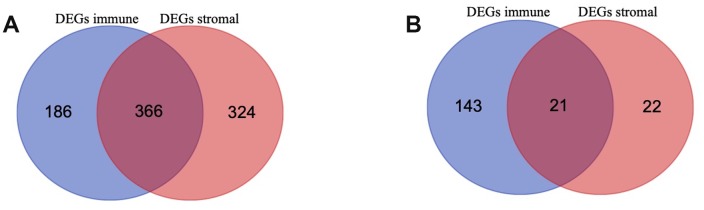
Differentially Expressed Genes (DEGs) selected (**A**, **B**) Venn diagram of differentially expressed genes (DEGs) base on immune and stromal score. (**A**) shows the commonly upregulated DEGs and (**B**) shows the commonly downregulated DEGs.

To identify the immune-related and stromal-related genes, differential analysis by using “limma” package was performed (Supplementary Table 3). 552 genes were upregulated in the high immune score group (purple circle in Figure 3A), and 690 genes were upregulated in the high stromal score group (red circle in Figure 3A). At the same time, 164 genes were downregulated in the high immune score group (purple circle in Figure 3B), and 43 genes were downregulated in the high stromal score group (red circle in Figure 3B). 387 intersection genes were selected for further analysis (overlap zone in Figure 3A, 3B).

### Enrichment analysis of intersection genes

**Figure 4:**
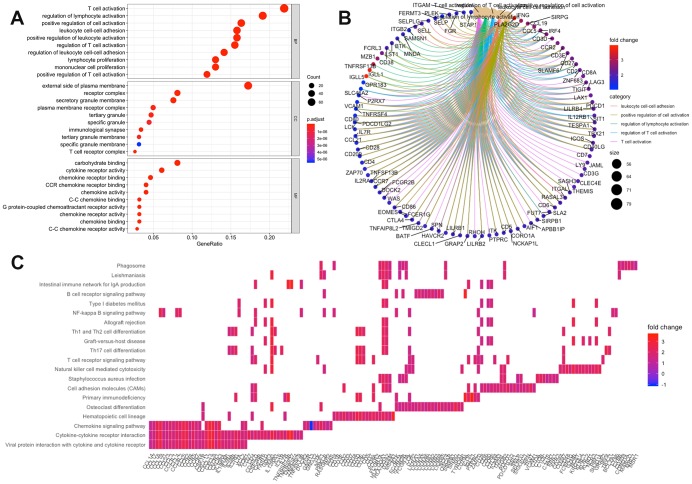
**Enrichment analysis of microenvironment related differentially expressed genes (DEGs).** (**A**) the top 10 of biological processes GO terms, cellular component GO terms, molecular function GO terms; (**B**) The correlation between intersection genes and top 5 biological processes GO terms; (**C**) KEGG (Kyoto Encyclopedia of Genes and Genomes) analysis of immune-related DEGs.

Using the “clusterProfiler” R package, 711 Gene Ontology (GO) terms and 36 Kyoto Encyclopedia of Genes and Genomes (KEGG) terms were indicated (Supplementary Table 4). The results showed the top 10 biological processes GO terms, cellular component GO terms, molecular function GO terms (Figure 4A), and the top 20 KEGG pathway terms (Figure 4C). The correlation between the intersection genes and the top 5 biological processes, including T cell activation, regulation of lymphocyte activation, regulation of T cell activation, leukocyte adhesion, and positive regulation of cell activation is shown in Figure 4B. The KEGG analysis showed that the intersection genes were associated with immune responses, especially T cell responses.

### Analysis of protein-protein interactions (PPI) among intersection genes

**Figure 5:**
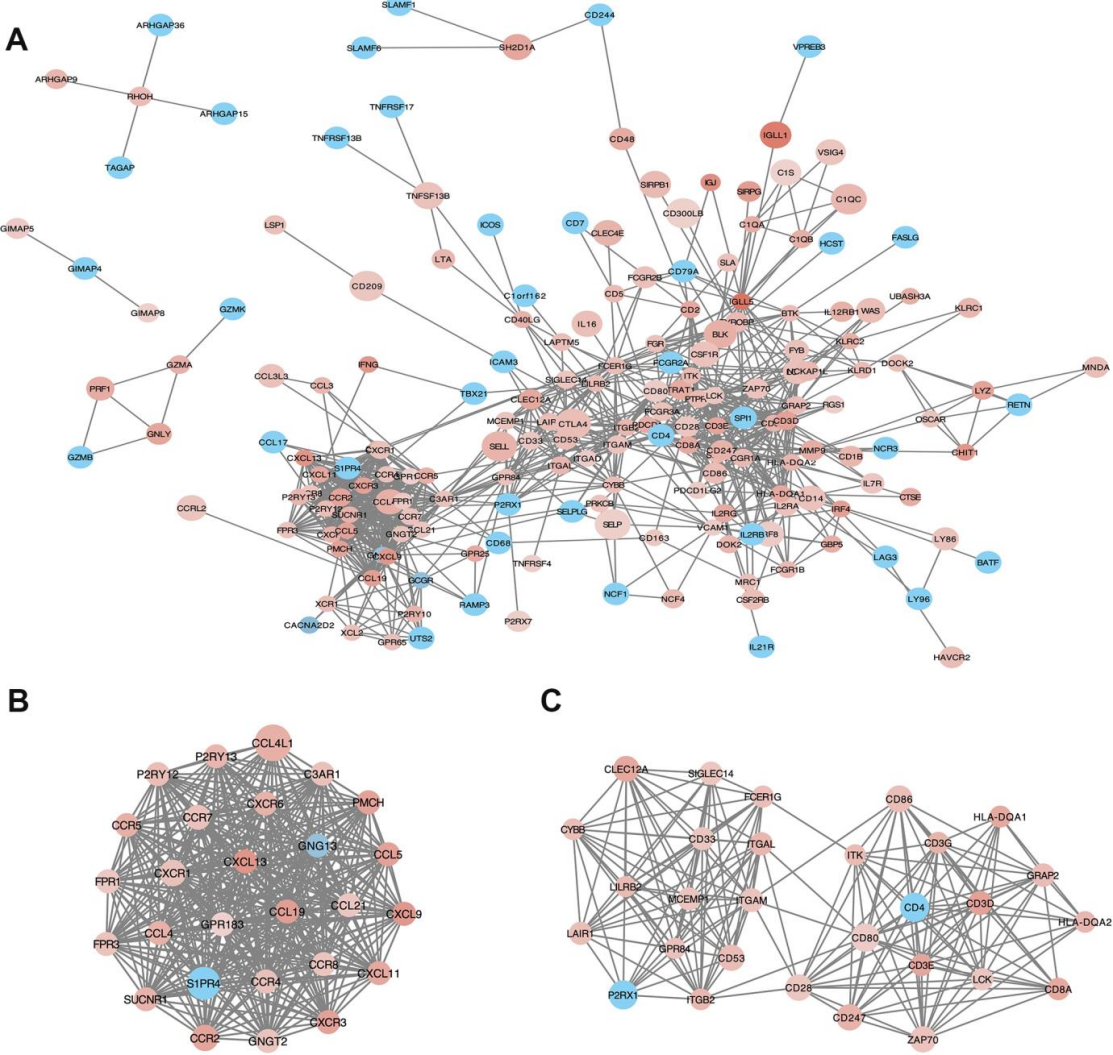
**Protein-protein interaction (PPI) network of microenvironment related genes.** (**B**) module 1 and (**C**) module 2 are the top two modules (>10 nodes) in the PPI network (**A**). The color of nodes associate with the log(FC) value, and the size reflects the combine score.

PPI network with 384 nodes and 1784 edges was constructed using the STRING website (Figure 5A). Using the MCODE software we found modules in the network; modules including at least 10 nodes were selected (Figure 5B, Module 1; Figure 5C, Module 2). GO and KEGG analyses of module 1 (Figure 5B) by ClueGo are shown in Supplementary Figure 2A and 2C. Likewise, GO and KEGG analyses of module 2 (Figure 5C) by ClueGo are shown in Supplementary Figure 2B and 2D. The results demonstrated that the module 1 was mainly enriched in leukocyte migration (59.84%; Supplementary Figure 2A), Toll-like receptor signaling pathway (40%), and chemokine signaling pathway (40%; Supplementary Figure 2C). Module 2 was mainly enriched in T cell co-stimulation (72.28%; Supplementary Figure 2B) and cell adhesion molecules (40.54%; Supplementary Figure 2D).

### Identification of TME associated genes based on machine learning

**Figure 6:**
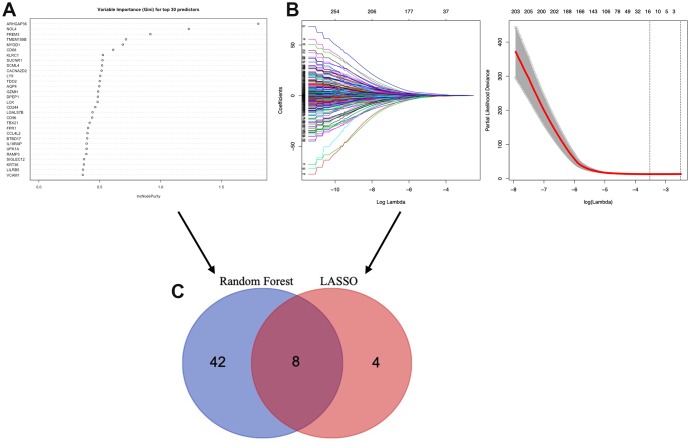
**Selection of microenvironment related prognostic genes.** (**A**) Random forest and (**B**) Lasso (Least Absolute Shrinkage and Selector Operation) algorithms were preformed to further select microenvironment related prognostic genes. (**C**) Venn diagram analysis between the genes selected by Random forest algorithm and Lasso algorithms.

To identify the TME associated genes, we performed two different machine learning algorithms, LASSO algorithm and Random forest algorithm. By LASSO algorithm, 12 genes were identified (Figure 6B); by Random forest algorithm, 50 genes were identified (Figure 6A). The ROC curve of LASSO (Supplementary Figure 1A, AUC:0.753) and Random forest test (Supplementary Figure 1B, AUC:0.960) was used. An overlap between the above two groups identified 8 TME associated genes (AQP4, ARHGAP36, CACNA2D2, CTSW, NOL4, SIGLEC1, TMEM150B and TRPM5) (Figure 6C).

### Predictive signature construction and survival analysis

**Figure 7:**
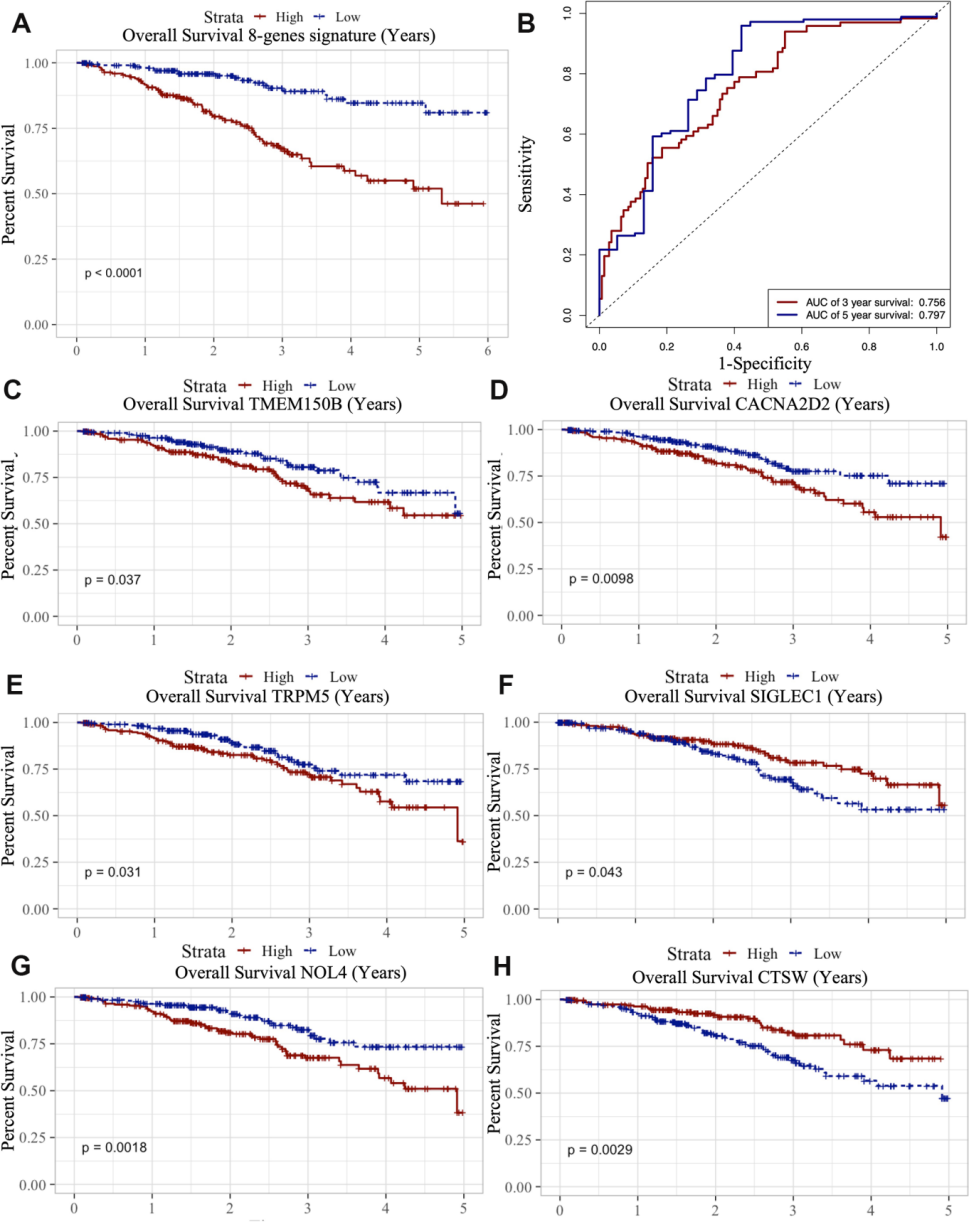
**Survival analysis of microenvironment related prognostic genes.** (**A**) Kaplan-Meier (KM) survival curve of 8 microenvironment related prognostic signature. (**B**) ROC (receiver operating characteristic) curve of 8 microenvironment related prognostic signature. (**C**–**G**) Kaplan-Meier (KM) survival curve of microenvironment related prognostic genes.

Multivariate Cox regression analysis performed by “survival” R package was subsequently used to construct a predictive signature using the above 8 TME associated genes. The risk score of the prognostic signature was then calculated according to the formula: $\text{risk score}=\sum_{i=1}^{n}\beta i*xi$ (β stands for the regression coefficient) [15]. By Kaplan-Meier survival analysis, we found that this 8-gene signature was associated with OS (*p*<0.0001) (Figure 7A); this was validated by the ROC curve (AUC of 3 year: 0.756, AUC of 5 year: 0.797) (Figure 7B).

We also analyzed the association between the 8 genes and OS using the Kaplan-Meier survival analysis. We found that the high levels of TMEM150B (*p*=0.037), CACNA2D2 (*p*=0.0098), TRPM5 (*p*=0.031) and NOL4 (*p*=0.0018) negatively correlated with OS (Figure 7C–7G), while the high levels of CTSW (*p*=0.0029) and SIGLEC1 (*p*=0.043) positively correlated with OS (Figure 7H).

### Immune cells infiltration analysis

**Figure 8:**
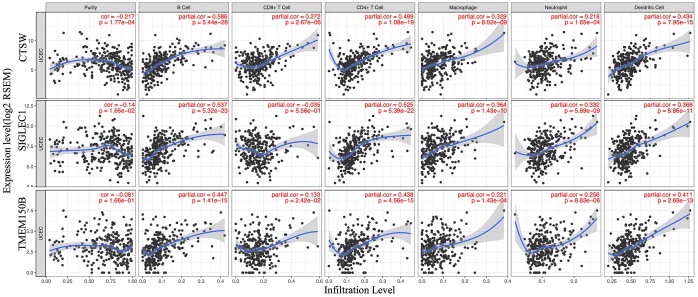
**Correlation of microenvironment related prognostic genes’ expression with immune infiltration level.**

To determine whether there is a correlation between tumor infiltration with immune cells, and immune-related gene expression, the tumor infiltration with six types of immune cells (CD4 + T cells, CD8 + T cells, B cells, neutrophils, macrophages, and dendritic cells) was analyzed by TIMER (Supplementary Table 5). Figure 8 shows the correlation between the immune cell infiltration and the expression of prognostic genes. The expression of CTSW positively correlated with the infiltrating levels of B cells (partial.cor=0.586, *p*=5.44e-28), CD4+ T cells (partial.cor=0.499, *p*=1.08e-19), macrophages (partial.cor=0.329, *p*=8.02e-09), and dendritic cells (partial.cor=0.434, *p*=7.95e-15). SIGLEC1 was associated with infiltrating levels of B cells (partial.cor=0.537, *p*=5.322e-23), CD4+ T cells (partial.cor=0.525, *p*=5.39e-22), macrophages (partial.cor =0.364, *p*=1.43e-10), neutrophils (partial.cor=0.332, *p*=5.89e-09), and dendritic cells (partial.cor=0.368, *p*=8.66e-11). Similarly, TMEM150B was associated with infiltrating levels of B cells (partial.cor=0.447, *p*=1.41e-15), CD4+ T cells (partial.cor=0.438, *p*=4.56e-15), and dendritic cells (partial.cor=0.411, *p*=2.68e-13) (Figure 8).

## DISCUSSION

Tumor microenvironment (TME) plays a critical role in tumor development, progression, and responses to therapies, especially immunotherapies. However, the role of TME differs in different types of cancer. Although endometrial cancer is one of the most common gynecological cancers, the composition of the TME, and its correlation with EC prognosis remain poorly understood compared to other malignancies.

In this study, we performed a comprehensive analysis of immune cells and genes in the TME of endometrial carcinoma, and related the data to clinical outcomes and prognosis of EC patients. Using the ESTIMATE algorithm, we first analyzed the correlation between the immune/stromal scores and the clinical EC characteristics obtained from TCGA-CDR. The ESTIMATE algorithm is a widely accepted and reliable algorithm that has been used in various cancers, including glioblastoma [16], breast cancer [17], prostate cancer [18], colon cancer [19], and cutaneous melanoma [20]. Using the ESTIMATE algorithm, our data demonstrate that the immune and stromal scores positively correlate with clinical outcomes of EC patients.

The pathogenetic types of endometrial cancer include endometrioid endometrial adenocarcinoma, serous endometrial adenocarcinoma, and mixed serous and endometrioid endometrial adenocarcinoma. Patients with serous endometrial adenocarcinoma often have a relatively poor prognosis [21–23]. In our study, we found that the immune and stromal scores of endometrioid endometrial adenocarcinoma were significantly higher than in serous endometrial adenocarcinoma, suggesting that the high concentration of immune cells in the TME of endometrioid endometrial adenocarcinoma might represent one of the mechanisms contributing to the good prognosis of this type of EC cancer. In addition, by analyzing the correlation between the immune scores and tumor recurrence, our data show that high immune score patients have longer progression-free interval and overall survival rates, indicating that the TME composition affects the clinical outcomes of EC patients.

Next, we analyzed differentially expressed genes (DEGs) in EC, by dividing patients into high score and low score groups, based on the median immune/stromal scores. Our data show that DEGs are involved in TME, and specifically regulate T cell functions. Furthermore, analysis of the PPI network indicated enrichment clustered in T cell functions, including T cell migration, differentiation, co-stimulation, and receptor signaling. We speculate that these TME associated genes might affect the development of endometrial cancer by affecting the T cell functions.

Because of its accuracy and predictive performance, the machine learning algorithm has been used in different fields, including medical diagnostics [10].

Since complex models and highly significant relationships can be extracted from large amounts of data, the machine learning can be highly predictive for specific cancers [24, 25]. LASSO algorithm [26] and Random forest (RF) algorithm [27] can be used for classification and regression; thus, they were particularly well-suited in our study to identify the prognostic TME-related genes. Using this approach, eight immune-related genes were identified; high levels of TMEM150B, CACNA2D2, TRPM5, and NOL4 showed a negative correlation to OS, while high levels of CTSW and SIGLEC1 showed a positive correlation to OS in EC patients. These genes have been reported to be involved in carcinogenesis and development of various cancers. TMEM150B regulates autophagy and cell death by encoding an autophagy regulator [28]. CACNA2D2, the auxiliary subunit of α2δ2, induces cell proliferation and angiogenesis by increasing the expression of vascular endothelial growth factor to promote tumorigenesis [29]. NOL4 (nucleolar protein 4) is a novel methylation target in cervical cancer, and has been suggested as an early detection and risk prediction biomarker in cervical cancer [30]. However, most of the identified genes have not been previously linked to EC. Our data indicate that they could serve as potential prognostic biomarkers for EC.

To investigate the impact of TME infiltration with immune cells on the prognosis of EC patients, we calculated the degree of infiltration of six immune cell types by using TIMER algorithm, and correlated the data with the expression of the identified immune genes. SIGLEC1 (CD169) is a novel biomarker of tumor-associated macrophages [24]. A previous study found that the density of CD169+ macrophages was positively associated with the abundance of CD8(+) CTL and CD57(+) NK cells in tumor tissues, and correlated with a better prognosis in EC patients [31]. This finding is consistent with the results obtained in our study. CTSW (Cathepsin W) is a novel human cysteine protease expressed in CD8+ T cells and NK cells [32], and plays an important role in cellular cytotoxicity mediated by NK cells and CD8+ T cells [33, 34]. Different T cell populations have different functions in regulating tumor grade, stage, and invasion ability in endometrial cancer [35–37]. We speculate that CTSW might be involved in the development of EC by regulating the T cell functions.

Previous studies have suggested that the tumor microenvironment in EC may have a significant prognostic value, and even play a role in resistance to treatment [38–40]. However, the tumor microenvironment is complex, and is determined by many factors. To improve the accuracy and reliability of the TME analysis, we used a large global collection of EC tissues from TCGA-UCEC, and introduced two machine learning algorithms. In addition, this study comprehensively analyzed the correlation between microenvironmental and genetic factors, and identified six potential prognostic TME-related genes (CACNA2D2, CTSW, NOL4, SIGLEC1, TMEM150B, and TRPM5). Future studies should identify the specific roles these genes play in the regulation of EC development and progression.

## MATERIALS AND METHODS

R software (version 3.5.1) [41] and Bioconuctor [42] were used for all analyses in this study.

### Data collection and analysis

All RNA expression data were obtained from The Cancer Genome Atlas (TCGA) (Data Release 16.0 - March 26, 2019) (https://portal.gdc.cancer.gov/). The expression data were then normalized by Fragments Per Kilobase of transcript per Million mapped reads (FPKM). The corresponding clinical data were obtained from TCGA-CDR (TCGA Pan-cancer Clinical Data Resource) dataset [14]. Patients whose overall survival times or progression-free interval (PFI) times were less than 30 days were excluded from our study. Progression-free interval is characterized as a time without a new tumor occurrence or a death from cancer. In total, data from 521 patients were analyzed in our study, and 19459 RNAs were extracted from RNA-seq data according to ENSEMBL Genomes (hg38) (http://ensemblgenomes.org/). Both RNA-seq data and corresponding clinical data were publicly available.

### Calculation of immune and stromal scores

ESTIMATE (Estimation of STromal and Immune cells in MAlignant Tumor tissues using Expression data) is one of the algorithms developed to evaluate the cell tumor composition by calculating the immune and stromal scores using Pearson’s correlation coefficient [6]. By using “estimate” R package, the immune and stromal scores were calculated based on the gene expression data of EC patients.

### Selection of differentially expressed genes

The samples were divided into high and low immune/stromal score groups based on the medium values of the immune/stromal scores. The selection of differentially expressed genes (DEGs) was performed by using “limma” R package with p-value < 0.05 and log fold change > 1 as a filter [43]. A website Venn diagrams tool (http://bioinformatics.psb.ugent.be/webtools/Venn/) was used to identify the commonly upregulated or downregulated DEGs in the immune and stromal groups. These intersection genes were selected for further analysis.

### Enrichment analysis of intersection genes

GO (Gene Ontology) and KEGG (Kyoto Encyclopedia of Genes and Genomes) enrichment analyses and visualization of intersection genes were performed by “clusterProfiler” R package [44] and “enrichplot” R package [45] with p-value < 0.05 as the cut-off value.

### Protein-protein interaction (PPI) network construction

For understanding protein interactions, we constructed the PPI network by STRING (V11) [46] with high confidence (0.7). The information of nodes of the PPI network was then further analyzed by Cytoscape software [47]. In Cytoscape, we used Molecular COmplex DEtection (MCODE) to select clusters which included 10 or more nodes [48]. ClueGo App was used to perform enrichment analysis of each cluster selected by MCODE [49].

### Identification of TME associated prognostic genes

Least Absolute Shrinkage and Selector Operation (LASSO) algorithm was used to identify candidate genes by “glmnet” R package with number of lambda = 1000 [50]. Clinical outcomes and gene expression profiles were analyzed by LASSO and Random forest algorithms. Lambda.min is the cutoff point that brings minimum mean cross-validated error. Genes with the highest lambda values were selected for further analysis. Simultaneously, Radom Forest algorithm was used for candidate genes selection by “randomForest” R package [51]. According to “randomForest” package, we set “ntree” as 10001 and “mtry” as a default value. No other options were used in machine learning algorithm.

The overlapped genes in LASSO and Random Forest algorithms were selected and then "survival" R package [52] was used to preform multivariate cox regression. The ROC (time-dependent receiver operating characteristic) curve was used to estimate the accuracy and specificity of the classification performance of the gene signature.

### Overall survival analysis

Overall survival analysis was performed by Kaplan-Meier survival analysis by using “survival” R package [52], overall survival times of EC patients (n=521), and gene expression data.

### Analysis of immune cell infiltration

The TIMER (Tumor IMmune Estimation Resource) algorithm was used to calculate the tumor abundance of six infiltrating immune cells (CD4 + T cells, CD8 + T cells, B cells, neutrophils, macrophages, and dendritic cells) based on RNA-Seq expression profiles data [12]. Compared to other calculation methods, the TIMER algorithm can eliminate bias effects by removing highly expressed genes and eliminating collinearity between immune cells to ensure accuracy of the calculation.

The correlation between the selected prognostic genes and immune cells was calculated by Spearman’s correlation analysis by TIMER. The correlation coefficient value<0.3 indicates that the correlation is negligible, while the correlation coefficient >0.3 indicates a positive/negative correlation [53].

## Supplementary Material

Supplementary Figures

Supplementary Table 1

Supplementary Table 2

Supplementary Table 3

Supplementary Table 4

Supplementary Table 5
