# Supplementary material for: Identification of prognostic immune-related genes in the tumor microenvironment of endometrial cancer
Source: Aging (Albany NY). 2020 Feb 19;12(4):3371–87. doi: 10.18632/aging.102817 (PMC7066904; doi:10.18632/aging.102817)
Supplement: Supplementary Figures [file aging-12-102817-s006..pdf]

SUPPLEMENTARY FIGURES

Supplementary Figures

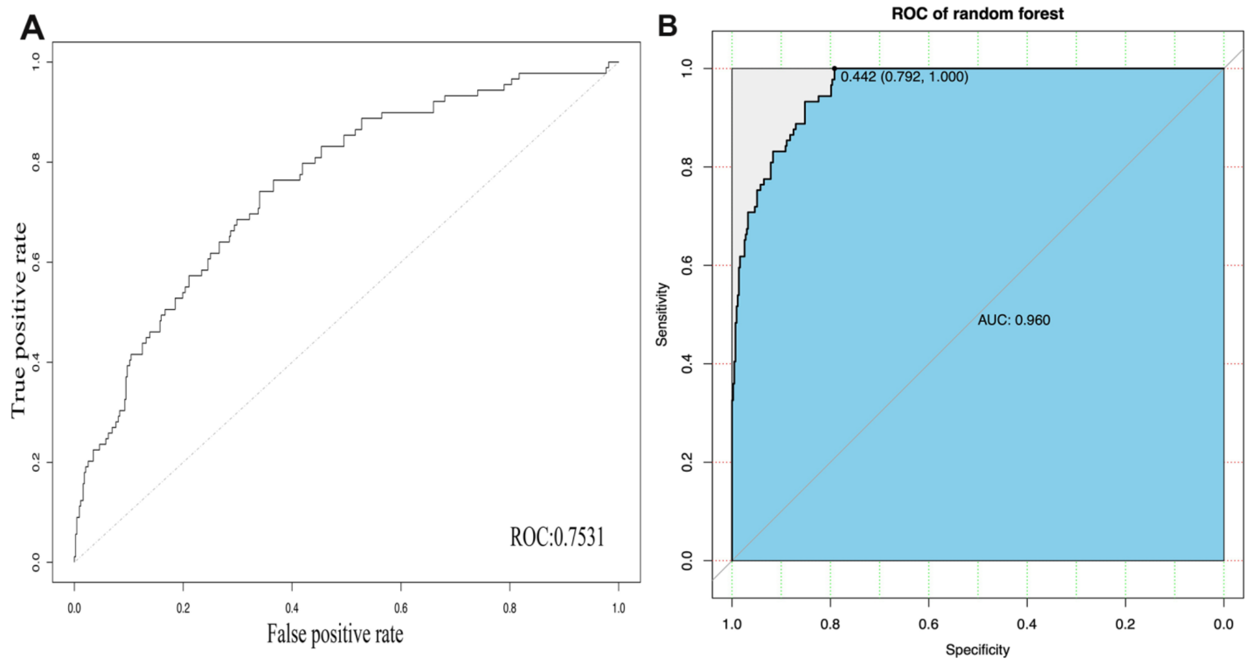

Supplementary Figure 1. ROC (receiver operating characteristic) curve of LASSO algorithm (**A**) and Random forest algorithm (**B**).

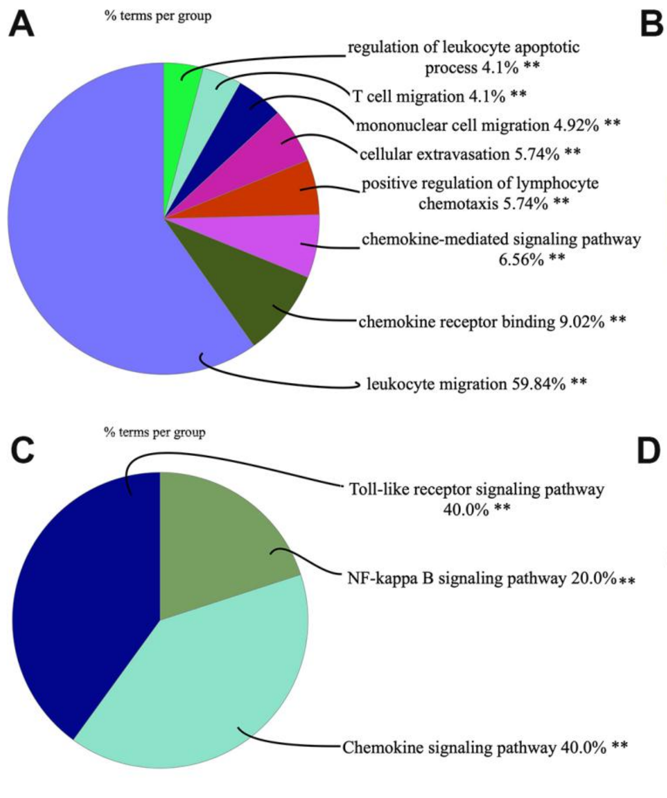

Supplementary Figure 2. Functional enrichment of key modules of PPI network. GO analysis (**A**) and KEGG (**C**) analysis of module 1. GO analysis (**B**) and KEGG (**D**) analysis of module 2
